# Supplementary material for: A Global Screen for Assembly State Changes of the Mitotic Proteome by SEC-SWATH-MS
Source: Cell Syst. 2020 Feb 26;10(2):133–155.e6. doi: 10.1016/j.cels.2020.01.001 (PMC7042714; doi:10.1016/j.cels.2020.01.001)

P61009 | SPCS3_HUMAN | SPCS3 SPC22 UNQ1841/PRO3567
Monomer MW [kDa]: 20.313 Monomer expected elution fraction: 54

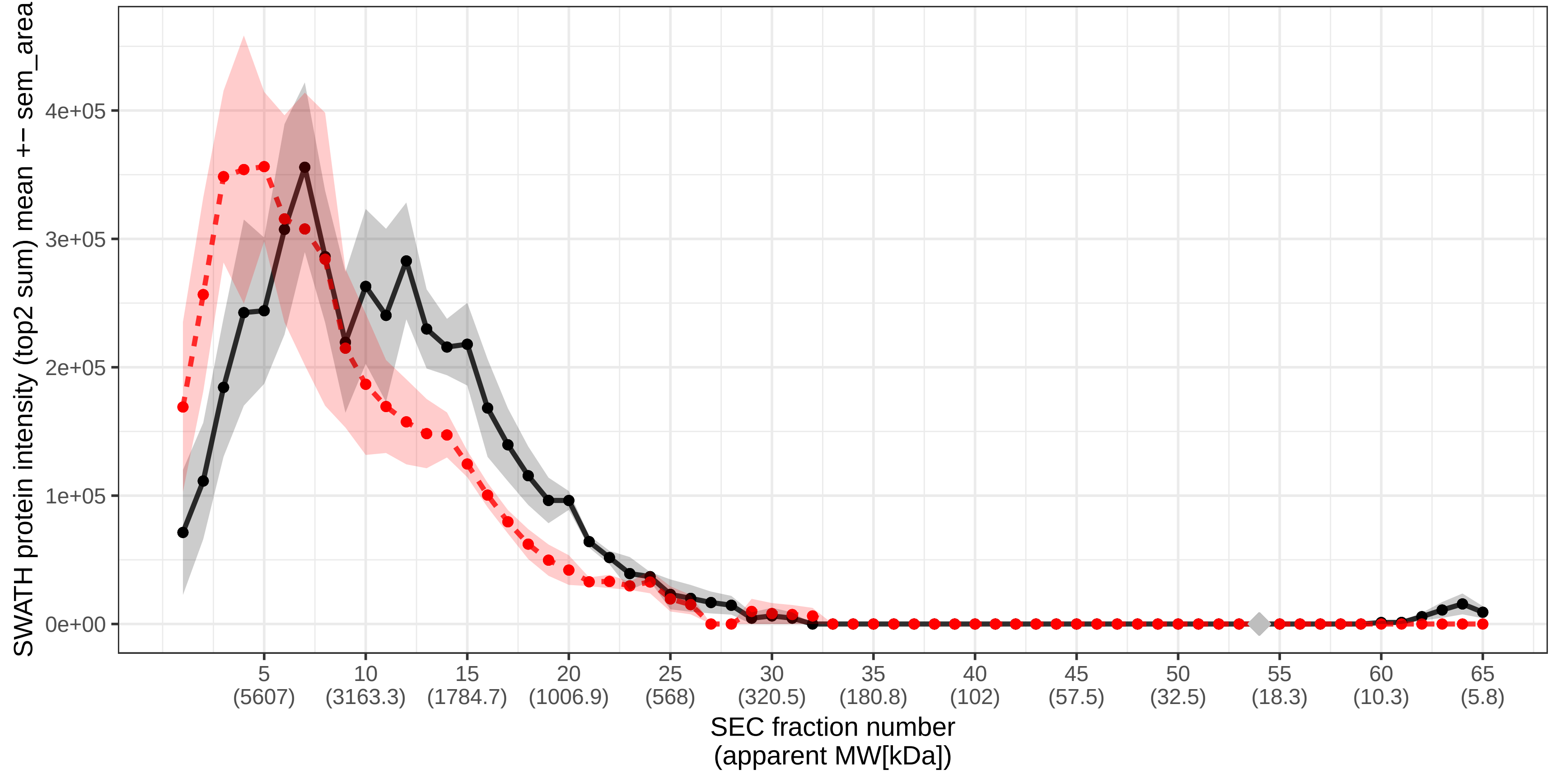

Supplement: Data S1. SEC-SWATH-MS Protein Chromatograms, Related to Figure 1 [file mmc6.zip › SECchrom_P61009_SPCS3_HUMAN_SPCS3_SPC22_UNQ1841_PRO3567.pdf]
